# Chrono-combined aerobic-resistance exercises as therapeutic approach to reverse neurodegeneration in rat model: a detailed protocol

**DOI:** 10.3389/fnagi.2026.1775321

**Published:** 2026-04-29

**Authors:** Muhammad Hafiz Zuhdi Fairof, Hussin Muhammad, Amirul Hafiz Ahmad Abdullah, Farah Wahida Ibrahim, Theng Choon Ooi, Nur Liana Md Nasir, Chin Long Poo, Siti Soleha Ab Dullah, Mohd Rahimi Ashraf Abd Rahman, Elda Nurafnie Ibnu Rasid, Arimi Fitri Mat Ludin, Nor Fadilah Rajab

**Affiliations:** 1 Centre for Healthy Ageing and Wellness (H-CARE), Faculty of Health Sciences, UKM, Kuala Lumpur, Malaysia; 2 Toxicology and Pharmacology Unit, Herbal Medicine Research Centre, Institute for Medical Research, National Institutes of Health, Ministry of Health, Setia Alam, Malaysia; 3 Faculty of Health Sciences, Center for Toxicology and Health Risk Studies (CORE), UKM, Kuala Lumpur, Malaysia; 4 Premier Integrated Labs Sdn. Bhd, Kuala Lumpur, Malaysia

**Keywords:** chrono-exercise, combined aerobic-resistance exercise, muscle-brain crosstalk, neurodegenerative, neuroprotection

## Abstract

The global increase in neurodegenerative disorders such as Alzheimer’s disease has prompted the search for effective non-pharmacological interventions. Chrono-exercise which is the physical training aligned with circadian rhythms has emerged as a novel strategy to strengthen cognitive resilience. This study explores the impact of chrono-exercises, incorporating aerobic, resistance, and combined modalities, performed at the early dark (ZT13) and early light (ZT1) phases in an aluminum chloride (AlCl_3_)-induced rat model of neurodegeneration. One hundred male Wistar rats aged 2–3 months will undergo structured exercise interventions following AlCl_3_ exposure. Neurobehavioural assessments including novel object recognition and open-field tests along with grip strength analysis, biochemical profiling and histological evaluations of brain and muscle tissues will be conducted. We hypothesize that the timing of combined aerobic-resistance exercise critically influences neuroplasticity and cognitive performance. Findings are expected to guide circadian-based exercise prescriptions to counter cognitive decline and enhance brain health in aging populations.

## Introduction

The global shift toward aging populations is a defining demographic challenge of the 21st century (United Nation, 2019). Malaysia officially became an aging nation in 2020, when individuals aged 60 and above made up 7.0% of the population. This figure is expected to more than double to 15% by 2030 (Mutalib et al., 2020; Sooryanarayana and Sazlina, 2020; Tey et al., 2016). This demographic shift driven by declining fertility rates and increasing life expectancy is particularly pronounced in developing countries. While the mortality due to infectious diseases is reduced, the burden of non-communicable diseases (NCDs), such as frailty, sarcopenia and neurodegeneration, is increasing due to prolonged life expectancy (Jia et al., 2022; Sinha and Ellis, 2021).

Cognitive decline progresses along a continuous spectrum from normal aging to mild cognitive impairment (MCI) and eventually to dementia. Although MCI may be reversible, dementia often results in irreversible loss of autonomy. The pooled global prevalence of mild cognitive impairment (MCI) is currently estimated at 19.7%, with a statistically significant increase observed in studies conducted after 2019 (Song et al., 2023). Based on recent evidence, the pooled prevalence of mild cognitive impairment (MCI) among older adults in the African region alone was estimated at 29.39% (Asnakew et al., 2025). In Malaysia, advancing age, sex, education level, social support, nutritional status, depressive symptoms and lifestyle factors, such as physical and cognitive engagement, have been identified as significant risk factors (Sugimoto et al., 2022). At the physiological level, chronic inflammation, oxidative stress and mitochondrial dysfunction are key contributors to the neurodegeneration.

Physical exercise has been increasingly recognized as a non-pharmacological intervention capable of mitigating cognitive decline. It enhances neuroplasticity, promotes cerebral angiogenesis and improves cerebral blood flow, collectively supporting brain function (Di Liegro et al., 2019; Scisciola et al., 2021). Previous study demonstrated that multicomponent exercise programs that incorporated endurance, resistance, flexibility and balance training were considered particularly effective in preserving both cognitive and functional capacities (Casas-Herrero et al., 2019). Additionally, physical exercise is associated with a lower risk of Alzheimer’s disease and other neurodegenerative conditions, highlighting its potential as a preventive strategy (Pahlavani, 2023).

Additionally, emerging research highlights the role of exercise timing in optimizing cognitive outcomes. Physiological responses, including strength and oxidative capacity vary across the circadian cycle, implying that the time of day when exercise is performed may influence its neuroprotective effects (van Moorsel et al., 2016). Furthermore, both chronotype and physical exercise have been independently linked to dementia risk, reinforcing the need to examine their interactions (Thapa et al., 2020).

Given these insights, the present study aims to investigate the neuroprotective effects of different exercises performed at different times of day using an AlCl_3_-induced neurodegenerative rat model. The investigation will comprehensively assess neurobehavioural performance, the expression of cognition- and exercise-related biomarkers, skeletal muscle function (via grip strength) and histological changes in the brain.

These findings are expected to aid in the tailored exercise prescriptions that incorporate optimal timing for individuals at risk of neurodegenerative disorders particularly in early-stage Alzheimer’s and Parkinson’s disease. By elucidating the association between circadian rhythms, brain function and exercise, this research contributes to the growing field of exercise chronobiology. As aging populations face an escalating burden of neurodegeneration, identifying cost-effective, non-invasive strategies to preserve cognitive health becomes increasingly vital.

Herein, we present a detailed experimental study protocol to evaluate the effects of chrono- combined aerobic-resistance exercise on synaptic plasticity, focusing on muscle-brain crosstalk in an AlCl_3_-induced neurodegeneration model. We aim to assess neurobehavioural changes, quantify relevant biomarker expressions, measure skeletal muscle strength and examine histopathological alterations in brain tissue to elucidate the therapeutic potential of time-optimized exercise interventions.

## Materials and analysis

### Experimental design

Male Wistar rats will be included in this study and randomly assigned to one of two primary groups: an AlCl_3_-treated group and a control group receiving distilled water (dH_2_O). The experimental period spans 8 weeks. Each primary group will then be subdivided into 10 subgroups (*n* = 10 each), based on varying exercise timing protocols and modalities. Aluminum chloride (AlCl_3_) was selected as a mechanistically focused neurotoxic model to induce oxidative stress and neuroinflammation-associated cognitive impairment, rather than to replicate the full clinicopathological spectrum of sporadic Alzheimer’s disease (AD). Although AD is multifactorial encompassing amyloid-β deposition, tau hyperphosphorylation, synaptic dysfunction, and network disconnection, oxidative stress and mitochondrial dysfunction are recognized as early convergent mechanisms interacting with these pathologies (Shen et al., 2023; Voigt et al., 2019). The AlCl_3_ exposure has been shown to promote amyloid-beta accumulation, tau hyperphosphorylation and synaptic loss, partially resembling AD-related features (Dial et al., 2024). AlCl_3_ reliably disrupts redox homeostasis through overproduction of reactive oxygen species (ROS), depletion of antioxidants, and impairment of the Nrf2 pathway, thereby driving neuroinflammation and neuronal vulnerability (Dial et al., 2024; Ehrlich et al., 2025; Sato et al., 2019). It also induces mitochondrial dysfunction including electron transport chain disruption, ATP depletion, and ROS amplification and impairs cholinergic neurotransmission, contributing to cognitive and motor deficits (Dial et al., 2024; Ehrlich et al., 2025; Sato et al., 2019). The administration of AlCl_3_ also produces hippocampal neuronal damage, microglial dystrophy, elevated IL-18, reduced IL-10, and increased markers of cellular senescence, collectively supporting a reproducible neurodegeneration-like phenotype (Mishima et al., 2025).

The choice of AlCl_3_ over transgenic AD models is deliberate. The primary objective of this protocol is to evaluate exercise-induced modulation of oxidative stress, mitochondrial integrity, and inflammatory signaling pathways, which are consistently disrupted by AlCl_3_ (Dial et al., 2024; Sato et al., 2019). The model offers controlled induction of pathology within a defined timeframe, cost-effectiveness, and alignment with the study’s mechanistic focus. While AlCl_3_ does not recapitulate the full complexity of sporadic AD and may involve dose- and route-dependent variability, the present study does not aim to generalize findings to Alzheimer’s disease as a whole. Instead, AlCl_3_ is employed as a reproducible model of oxidative stress–mediated neurodegeneration and neuroinflammation-associated cognitive impairment, representing a defined component of AD-related pathology (Dial et al., 2024). By administering AlCl_3_, this study aims to establish a neurodegenerative model to evaluate the therapeutic effects of exercise intervention.

The exercise intervention will overlap with ongoing AlCl_3_ exposure, reflecting a concurrent intervention model rather than a purely preventive approach. This design will allow evaluation of exercise effects on neurodegenerative processes during active degeneration, closely modeling real-world disease progression. AlCl_3_ administration will be initiated in the exercise intervention to ensure reliable establishment of the neurodegenerative model and activation of relevant pathological pathways, enabling assessment of exercise as a disease-modifying factor acting on an already initiated pathological cascade rather than as a pre-exposure protective strategy. By implementing exercise during continuous exposure to AlCl_3_, dynamic interactions between neurodegenerative stressors and neuroprotective adaptations can be investigated. The exposure parameters including dosage and duration will remain constant throughout the experiment.

This study also utilized Zeitgeber Time (ZT) to determine the exercise schedule, providing a standardized framework for studying the circadian influences on physiological responses. In chronobiology, ZT is a timekeeping system that aligns biological rhythms with environmental cues, primarily the light-dark cycle. Under this system, ZT 0 marks the onset of the light phase (lights on) (Shen et al., 2023; Voigt et al., 2019), while ZT 12 marks the onset of the dark phase (lights off) (Dial et al., 2024) with subsequent hours referenced accordingly. Several studies have highlighted that the timing of exercise is a critical determinant of both systemic and skeletal muscle–specific metabolic responses with exercise performed at different times of the day eliciting distinct metabolic and transcriptional outcomes (Ehrlich et al., 2025; Sato et al., 2019). Mishima et al. (2025) reported that plasma corticosterone concentrations were higher when resistance exercise was performed at ZT13 compared with ZT1, whereas the rate of muscle protein synthesis was greater following resistance exercise at ZT1 than at ZT13. Also, the purpose of selecting those time points (ZT1 and ZT13) was to investigate whether exercise-induced neuroprotective effects vary by circadian phase. Although circadian rhythms are known to regulate hormonal secretion, including corticosterone, as well as metabolic and oxidative processes, the present study did not directly assess hormonal fluctuations. Therefore, our interpretation focuses primarily on circadian phase–dependent physiological states rather than specific endocrine mechanisms. By adopting this pattern, we can investigate the time-dependent variations in exercise-related physiological and molecular processes. While rodents are nocturnal and humans are diurnal, translational chrono-exercise studies often model interventions relative to biological phase rather than strict light-phase equivalence. Specifically, this study focuses on biological responses at ZT 1 (1 h after lights on) and ZT 13 (1 h after lights off) as these time points allow us to compare early dark phase and early light phase effects corresponding to a post-active biological window relative to the preceding dark-phase activity. This approach enhances the understanding of circadian influences on the studied parameters providing insights into how exercise timing interacts with the body’s internal clock.

Groups 1–4 receive AlCl_3_ (200 mg/kg body weight) for 8 weeks and follow different evening exercise routines during the ZT 13 (Early Dark phase) period. Group 1 (Early Dark phase Sham) has no real exercise exertion, serving as a control. Group 2 (Early Dark phase Aerobic Exercise) performs treadmill running. Group 3 (Early Dark phase Resistance Exercise) does weight-loaded activities and Group 4 (Early Dark phase Combination Exercise) combines both aerobic and resistance exercises.

Groups 5–8 also receive AlCl_3_ for 8 weeks but exercise in the morning, ZT 1 (Early Light phase period). Group 5 (Early Light phase Sham) has no real exercise exertion as a control. Group 6 (Early Light phase Aerobic Exercise) does treadmill running. Group 7 (Early Light phase Resistance Exercise) performs weight-loaded activities and Group 8 (Early Light phase Combination Exercise) combines aerobic and resistance exercises.

Groups 9 and 10 serve as healthy controls without AlCl_3_ exposure. Both Group 9 (Non-treated + Early Dark phase Sham) and Group 10 (Non-treated + Early Light phase Sham) have no real exercise exertion in their respective activities but with the same handling procedure, apparatus placement, session duration and environmental conditions as the exercise groups. These groups serve as comparators to the effects of AlCl_3_ and exercise timing on neurodegeneration.

Sample size estimation was performed using G*Power (version 3.1) based on the primary behavioral endpoints, the novel object recognition test (NORT) and open field test (OFT), which serve as the outcomes driving the study’s statistical power. A repeated-measures ANOVA (within–between interaction) design was used for the *a priori* power analysis to model the expected interaction between intervention group and time (baseline, pre-intervention and post-intervention). The following parameters were specified: a medium effect size (*f* = 0.25; C *f* ohen), alpha (α) = 0.05, statistical power (1−β) = 0.95, number of groups = 10, number of repeated measurements = 3 and an assumed correlation among repeated measures of *r* = 0.5 with non-sphericity correction ε = 1. Based on these inputs, the required total sample size was estimated at 90 rats (nine per group). To account for potential attrition during the experimental period, an additional 10% contingency was included, resulting in a target sample size of 99 animals. For practical considerations and to ensure equal allocation across experimental groups, the final sample size was rounded to 100 rats (10 per group). This rounding does not materially increase statistical power but facilitates a balanced experimental design.

Secondary endpoints, including grip strength, plasma and brain biochemical markers and histological outcomes were not used in the initial sample size calculation. Histological analyses will be performed on a subset of animals (*n* = 3 per group) and will therefore be considered exploratory. For these analyses, multiple coronal sections per brain and multiple non-overlapping microscopic fields per section will be quantified. Importantly, the statistical unit of analysis will be the individual animal rather than individual sections, and measurements obtained from different sections will be averaged to generate a single value per animal. This approach reduces technical variability and is consistent with established neurohistological practices in experimental models of neurodegeneration (Chen et al., 2014; Hain et al., 2016; Liu et al., 2022).

**FIGURE 1:**
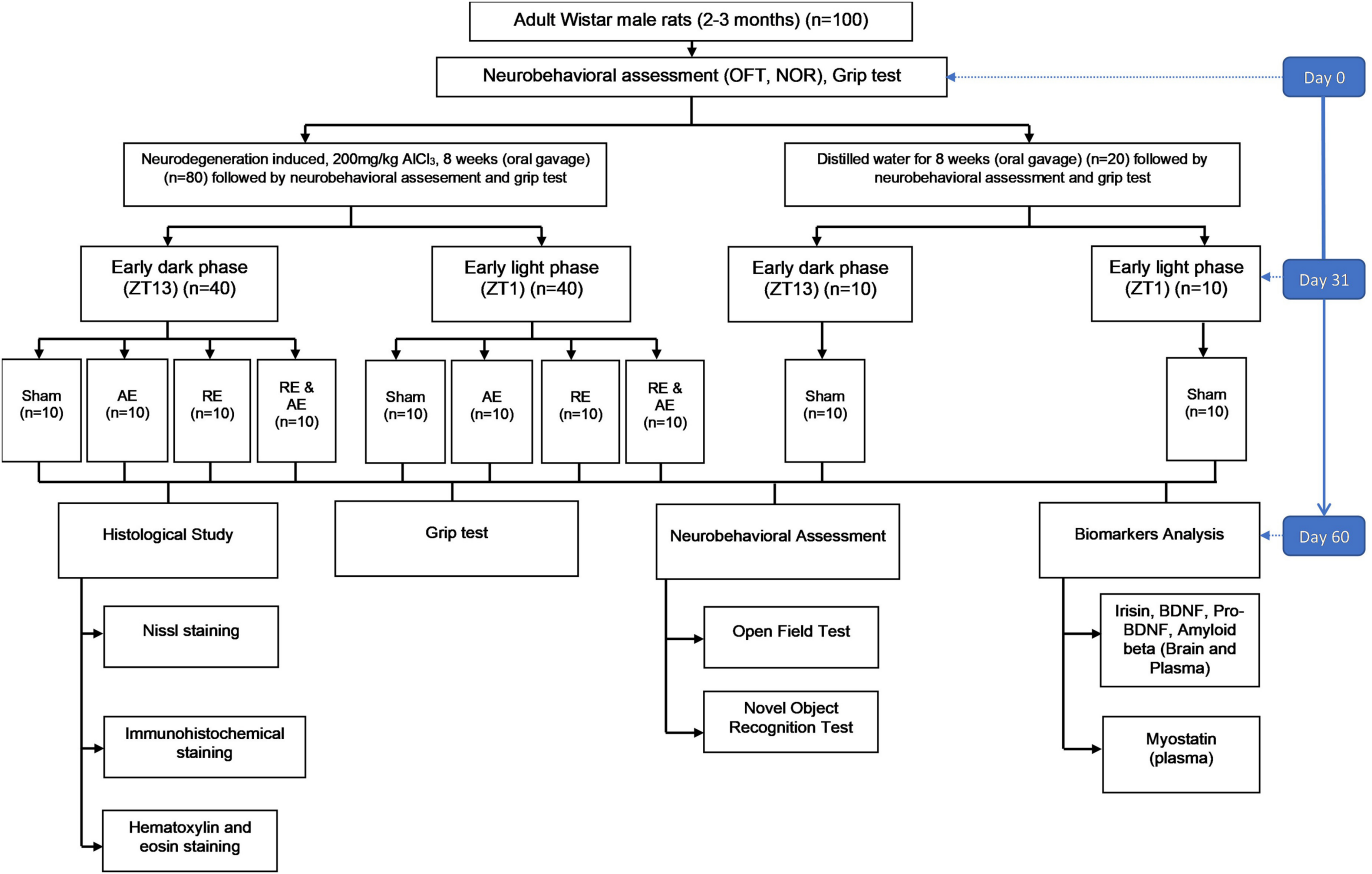
Schematic of the experimental design using adult male Wistar rats (*n* = 100) to assess different chrono-exercises effects on neurobehavioral, biochemical and histological outcomes in an AlCl_3_-induced neurodegeneration model.

Overall, the selected sample size is expected to provide adequate statistical power to detect meaningful differences in behavioral outcomes, grip strength and biomarker changes over time, consistent with established methodological recommendations for animal experimentation (Charan and Kantharia, 2013). A graphical overview of the experimental design is provided in Figure 1.

Daily treatments will be administered throughout the 8-week experimental period. The exercise intervention will commence after a 4-week AlCl_3_ induction phase. Behavioral assessments are conducted at three time points: baseline (pre-induction), pre-intervention and post-intervention (end of study). All results will be normalized relative to the pre-intervention time point to quantify intervention effects.

**FIGURE 2:**
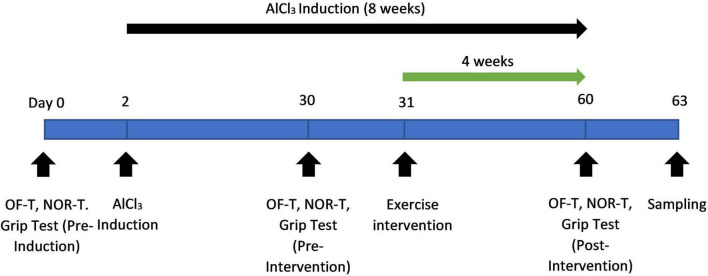
Timeline of the experimental protocol showing baseline testing, 8-week AlCl_3_ induction, 4-week exercise intervention and subsequent neurobehavioral assessments and biological sampling.

Following the final behavioral assessment, the rats will be euthanised using CO_2_ followed by decapitation for trunk blood collection 48 h after their last exercise session. This gap is to ensure the biological changes in the outcome measures are due to the chronic adaptation rather than acute responses (Benito et al., 2011; Laine et al., 2020; Li et al., 2020). Blood and brain tissues will be rapidly harvested to ensure the integrity of biochemical and histological analyses. Timely tissue isolation is essential to prevent post-mortem degradation and enable reliable examination of neurobiological outcomes. Figure 2 presents the experimental timeline while Table 1 outlines the complete schedule of interventions and assessments across the study period.

**TABLE 1:** Study schedule detailing key procedures across four phases.

Study procedure	Pre-induction	Pre-intervention	Intervention period	Post-induction
Days	0	2–30	31–60	61–63
Assessment				
Neurobehaviour				
NOR-T	X	X		X
OF-T	X	X		X
Muscle strength				
Grip test	X	X		X
AlCl3 treatment				
Induction (oral gavage)		X	X	
Exercise intervention				
Aerobic exercise			X	
Resistance exercise			X	
Combined exercise			X	
Sham exercise			X	
Sampling				
Sample extraction				X
Biomarkers analysis				
Irisin				X
BDNF				X
Pro-BDNF				X
Amyloid Beta				X
Myostatin				X
Histological analysis				
H&E staining				X
Nissl staining				X
Immunohistochemistry				X

### Experimental animal

Adult male Wistar rats (2–3 months old, 200–250 g) will be obtained from the Laboratory Animal Resource Unit Universiti Kebangsaan Malaysia following the approved ethical code FSK/2023/ARIMIFITRI/22-NOV-1383-DEC-2023-MARCH-2025. We will use only male rats to reduce variability related to hormonal cycles and to maintain consistency with previous studies in this model even though it is well-known that female rats with estrogen have been reported to modulate BDNF signaling and influence motor recovery outcomes (Ayala-Méndez et al., 2023; Becker et al., 2016; Dayton et al., 2016). They will be housed in pairs in standard polycarbonate cages with unrestricted access to food and water *(ad libitum)* and will be fed a standardized commercial chow to minimize dietary variability. All housing occurs in the Behavioral Laboratory, Faculty of Health Sciences, UKM, under controlled environmental conditions (22 °C–24 °C, 40%–60% humidity, 12-h light/dark cycle).

Before experimental procedures, all rats will undergo a 7-day acclimatization period. During this time, daily assessments of body weight and grooming behavior will be conducted to detect signs of chronic stress and minimize potential confounding effects on exercise-induced physiological responses.

Neurodegeneration will be induced using aluminum chloride (AlCl_3_), a widely used neurotoxicant that mimics Alzheimer’s disease-like pathology. Chronic AlCl_3_ exposure has been shown to trigger oxidative stress, neuroinflammation and tau protein hyperphosphorylation leading to neuronal damage and cognitive decline (Nasb et al., 2024). This model closely replicates key features of neurodegenerative disorders and is therefore suitable for evaluating the neuroprotective effects of targeted interventions.

### AlCl_3_ preparation and induction

The AlCl_3_ solution (20 mg/ml, reagent grade, sourced from Chemiz, Malaysia) (cat. no: 13338) will be prepared weekly by dissolving the compound in distilled water to ensure consistency and stability. The rats will receive a daily oral gavage of AlCl_3_ at a dose of 200 mg/kg body weight for 8 weeks, following a protocol previously established to induce neurodegenerative changes associated with cognitive decline (Ogunlade et al., 2022). To ensure accurate delivery, the indicated AlCl_3_ dose will be administered via oral gavage following standardized procedures conducted by trained personnel. The dosing solution will be freshly prepared, thoroughly mixed, and measured for each animal’s body weight using calibrated syringes. Appropriately sized gavage needles (16 gauge) will be employed to minimize spillage or regurgitation and animals will be monitored immediately after administration. Oral gavage will be used as a well-established method for precise oral dosing in toxicological research and will serve as a validated approach in aluminum exposure studies demonstrating consistent systemic uptake (Krause et al., 2020; Lim et al., 2022).

Daily injections will require repeated restraint and needle puncture which can cause significant stress, tissue injury and risk of infection in rodents potentially influencing physiological outcomes and confounding study results. Therefore, oral gavage will be selected as the most accurate, reproducible and ethically appropriate route for repeated aluminum administration. Throughout the induction period, the rats will be monitored for distress or adverse effects. Regular health assessments and common behavioral observations will be performed to ensure animal wellbeing and maintain study reliability

### Exercise intervention protocol

#### Aerobic exercise

**FIGURE 3:**
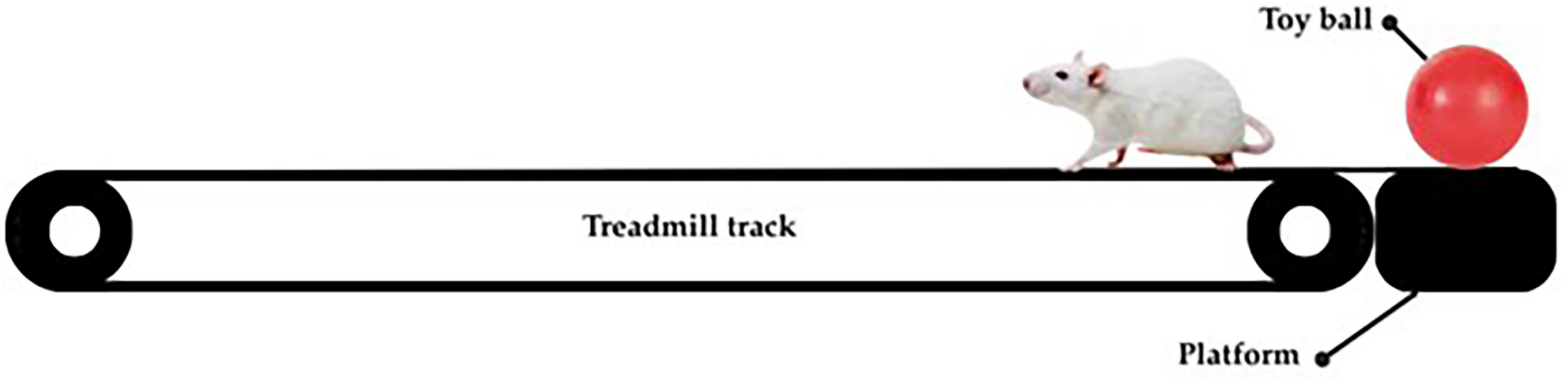
Illustration of aerobic exercise.

Aerobic training will be conducted 5 days per week and scheduled during either the early dark phase (ZT13) or the early light phase (ZT1) depending on group assignment. The protocol begins with a 3-day adaptation period during which treadmill speed is gradually increased (Sabouri et al., 2020). Each session will start with a 5-min warm-up at 5 m/min followed by a 25-min run at 8 m/min (Sabouri et al., 2020). This protocol with such intensity has been demonstrated to induce biological changes such as neuroplasticity improvement and modulation of oxidative stress and inflammation (Grabowska et al., 2025; Sabouri et al., 2020; Xu et al., 2021). A toy ball and gentle touch will be used as stimuli to encourage exercise engagement. Figure 3 illustrates the aerobic exercise intervention.

The rats will be subjected to resistance exercises using a custom device and a protocol developed by Al-Sarraf and Mouihate (2022). This exercise will involve training three times a week, on Mondays, Wednesdays, and Fridays accommodating both early light phase (ZT1) and early dark phase (ZT13) for the different group schedules. The protocol is designed to optimize outcomes, with each session incorporating 12–16 repetitions.

**FIGURE 4:**
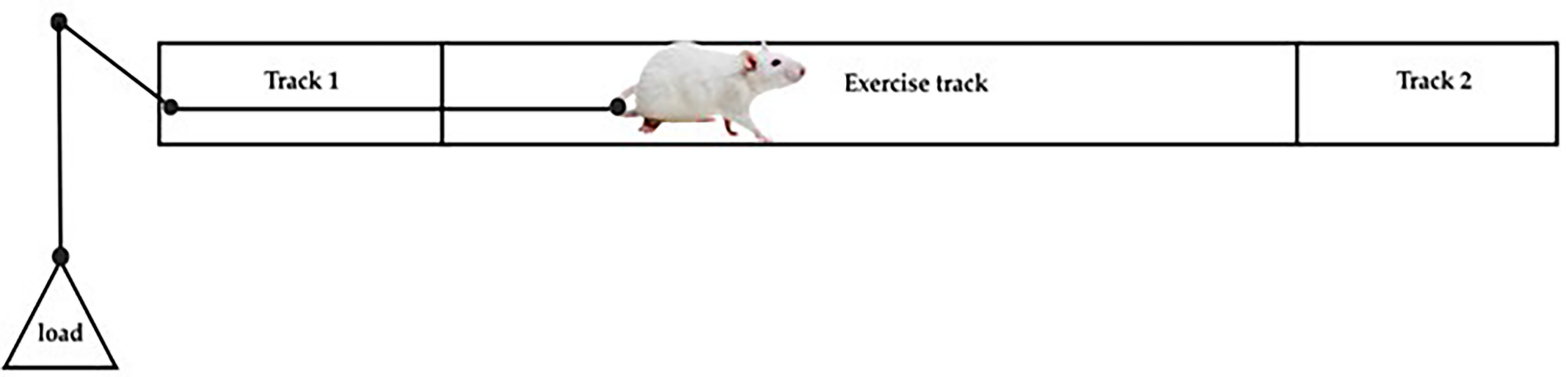
Illustration of resistance exercise.

Each training session will begin with four warm-up sets with no added weight as the initial load. The exercise intensity will progressively increase over those 4 weeks. In week 1, the load will be set at 25% of the rat’s body weight increased to 50%, 75%, and 100% in weeks 2, 3, and 4, respectively. This progressive overload protocol has been demonstrated in prior studies as an effective resistance training program for rodents to induce significant skeletal muscle hypertrophy, enhance muscle strength and modulate systemic metabolic parameters (Al-Sarraf and Mouihate, 2022; Wang et al., 2023). A gentle touch will be applied as a stimulus to encourage exercise engagement. Figure 4 illustrates the resistance exercise intervention.

#### Combination exercise (aerobic + resistance)

The combined protocol integrates both modalities alternating aerobic and resistance sessions over 5 days each week for 4 weeks (Al-Sarraf and Mouihate, 2022; Conti et al., 2015; Sabouri et al., 2020). Training is performed at the same circadian time either ZT1 or ZT13 as per the assigned group. The frequency is set at five sessions per week with the intensity varying according to the demands of the previous protocols for aerobic and resistance components.

### Sampling

#### Animal euthanasia using CO_2_

Euthanasia will be conducted using compressed CO_2_ gas in accordance with the American Veterinary Medical Association (AVMA) Guidelines for the Euthanasia of Animals (AVMA, 2020) and approved Institutional Animal Care and Use Committee (IACUC) protocols. Compressed CO_2_ will be delivered from a regulated cylinder directly into a euthanasia chamber ensuring precise control of flow rate. Rats will be introduced into the chamber without pre-charging and 100% CO_2_ will be administered at a displacement rate of 30%–70% of the chamber volume per minute which provides a gradual and humane rise in CO_2_ concentration (Stuckey et al., 2023). This approach will minimize distress and facilitate rapid unconsciousness typically within 2–3 min followed by respiratory arrest (Hickman, 2022).

CO_2_ flow will be maintained for at least one additional minute after cessation of respiration to ensure death. Each animal will be continuously observed throughout the procedure. If any signs of respiration, movement or reflexes persist, cervical dislocation will be performed as a secondary physical method (AVMA, 2019). Decapitation will be carried out immediately following CO_2_ euthanasia to ensure death and to facilitate rapid brain tissue collection. The use of CO_2_ euthanasia followed by decapitation does not compromise the structural integrity of neural tissue as prior studies have demonstrated that both gross and microscopic brain morphology remain intact and are comparable to those obtained through other humane methods (Ko et al., 2019).

The described procedure is consistent with AVMA guidelines which endorse CO_2_ inhalation followed by a secondary physical method such as decapitation (AVMA, 2019). This combined approach not only ensures complete euthanasia but also maintains high-quality tissue suitable for biochemical, histological and morphological analyses, thereby supporting both animal welfare and research integrity.

#### Trunk blood collection and processing

Following confirmation that the rats have been humanely euthanized, each animal will be placed on an absorbent-lined dissection tray. Decapitation will then be performed at the cervical level using sterile surgical scissors to facilitate trunk blood collection. Blood will be collected into EDTA-coated tubes and gently inverted 5–10 times to ensure adequate mixing with the anticoagulant.

Blood samples will be immediately placed on ice and transported to the laboratory for processing. Within 30 min of collection, blood tubes will be centrifuged at 4,000 rpm for 5–15 min s at 4 °C to separate plasma from cellular components (Haripersadh et al., 2022). Following centrifugation, the plasma will be carefully transferred into 1.5 mL microcentrifuge tubes using a pipette, ensuring no contamination with the buffy coat or red blood cells. Plasma will then be aliquoted into 200 μL portions in labeled microcentrifuge tubes to minimize freeze-thaw cycles during subsequent analyses. The aliquots will be stored immediately at −80 °C for biomarker analysis. To minimize the confounding effects of platelet lysis during plasma storage, plasma samples will be aliquoted after centrifugation to avoid repeated freeze-thaw cycles, which are known to exacerbate cellular and platelet lysis. In addition, storage at −80 °C helps to preserve structural damage to plasma components while the use of appropriate anticoagulants (EDTA) during plasma preparation helps stabilize labile analytes and prevent post-lysis degradation.

#### Organ harvesting and processing

##### Brain

After blood collection, the head will be positioned dorsally on a dissection tray immediately. Using fine surgical scissors and forceps, a midline incision will be made along the scalp from the nasal bone to the occipital region, exposing the skull. The skin and connective tissue will be carefully retracted laterally (Paxinos and Watson, 2014).

Using sharp dissecting scissors, the skull will be cut along the midline from the foramen magnum to the olfactory bulbs, followed by lateral cuts along the base of the skull to loosen the cranial plates. Fine forceps will then be used to gently peel back the skull, taking care to avoid damaging the underlying brain tissue. The whole brain will be carefully lifted from the cranial cavity using a spatula or fine forceps, ensuring the olfactory bulbs, cerebrum and brainstem remain intact. The harvested brain will be immediately placed on ice fixed in 4% PBS-buffered formalin and stored in a cold room for subsequent staining procedures.

The hippocampus and amygdala slices will be homogenized in 0.1 M phosphate buffer at pH 7.4 to prepare a 10% weight by volume (w/v) homogenate. Brain tissues will be homogenized at 12,000 rpm for 10 min at 4 °C using a tissue homogeniser (Li et al., 2023). It is well-known that proteases released during tissue homogenisation can degrade proteins and affect protein parameter measurements. To minimize protease activity, the temperature during sample processing will be set to 4 °C to limit enzymatic activity. Also, this process will be performed rapidly on samples to minimize the time available for protease activity before analysis. The supernatant will be used to quantify biochemical markers, which will be stored at −80 °C before analysis. All biological waste, including carcasses and blood-contaminated materials, will be disposed of in accordance with institutional biosafety regulations.

### Outcomes measure

#### Primary endpoint

The primary endpoint of this study is aligned to evaluate the therapeutic potential of the chrono-exercise interventions on muscle-brain crosstalk in the context of neurodegeneration. Specifically, alterations in neurobehavioural parameters, such as anxiety-related behaviors and non-spatial memory processing, will be assessed to determine how exercise types and timing modulate cognitive and muscular performance outcomes in AlCl_3_-induced neurodegeneration.

#### Behavioral analysis

##### Novel object recognition test (NOR-T) (non-spatial memory)

This test is designed to assess non-spatial memory performance by leveraging the natural exploratory behavior of rats, which have an innate tendency to explore novel objects more than familiar ones (Lueptow, 2017). The experiments will be conducted in soundproof rooms, illuminated by a red fluorescent light source exceeding 20 watts to ensure clear video recordings with a camera positioned above to capture the rats’ behavior. Before the trials, a 3-min familiarization period will be provided to allow the rats to acclimate to the testing environment. During the first trial, the rats will be allowed to explore two identical objects (two identical white glass bottles) (Reger et al., 2009) for 5 min. Following a predetermined intertrial interval, the second trial will introduce one familiar object from the first trial and one novel object (one darker glass bottle) (Reger et al., 2009) with a 6-min exploration period. Memory performance will be inferred based on the time spent investigating the novel object during the second trial.

A greater exploration time for the novel object compared to the familiar one suggests that the rat remembers the familiar object and is naturally drawn to investigate the new object. To quantify this memory performance, the Discrimination Index (DI) will be calculated using the formula:


D⁢I=(T⁢N-T⁢F)/(T⁢N+T⁢F)

Where TN represents the time spent exploring the novel object and TF represents the time spent exploring the familiar object. A higher DI value indicates better memory performance, providing a standardized measure of the rats’ ability to distinguish between familiar and novel objects (Antunes and Biala, 2012; Lueptow, 2017).

##### Open field test (OF-T) (anxiety-like behaviors)

The open field test (OF-T) will be used to assess exploratory behavior in novel environments and serve as an early screening tool for anxiety-related behaviors in rodents (Kraeuter et al., 2019). Since AlCl_3_-induced neurotoxicity is known to disrupt neurochemical pathways involved in anxiety regulation, this test will help determine whether chrono-exercise interventions can mitigate anxiety-like behaviors in AlCl_3_-treated rats. The experiments will be conducted under a red fluorescent light source exceeding 20 watts to ensure clear video recordings. Each rat will be placed at the edge of a black square arena (90 cm × 90 cm × 38 cm), which is divided into two zones: the peripheral zone and the central zone.

The rats’ activity will be recorded for 10 min using a camera positioned above the arena to capture their movements. Behavioral analysis will focus on scoring the frequency of entries into and crossings through the central zone, providing insight into the rats’ anxiety levels. More frequent entries into the central zone typically indicate lower anxiety, while avoidance of the central zone suggests heightened anxiety (Kraeuter et al., 2019; Seibenhener and Wooten, 2015).

##### Secondary endpoint

In addition to behavioral assessment, muscle strength testing will be conducted to confirm that the rats are physically capable of safely performing the prescribed exercise intervention and to ensure baseline functional comparability across groups. This assessment is not intended as a primary outcome measure of neuroprotection, but rather as a procedural and eligibility-verification step to support the feasibility and consistency of the chrono-exercises protocol. To elucidate the molecular mechanisms underlying exercise-induced neuroprotection, the study will further evaluate changes in exercise- and cognition-related biomarkers and cytokines. Biological samples will include blood plasma and brain homogenates obtained from the hippocampus and amygdala, enabling assessment of systemic and region-specific alterations associated with oxidative stress, inflammation, and neuroplasticity.

##### Grip strength test (muscle strength)

To assess muscle strength, the PowerLab system will be used comprising a grip strength meter with a force sensor, a data acquisition unit and LabChart software for real-time data collection and analysis. Before testing, the grip strength meter will be calibrated according to the manufacturer’s guidelines and connected to the PowerLab system via the appropriate analogue input channel. The LabChart program will be configured to collect force data from the designated channel, ensuring precise measurement of forelimb grip strength.

Each rat will be carefully handled to minimize stress before testing. The rat will be held by the base of its tail and allowed to voluntarily grasp the grip strength apparatus with its forelimbs. Once a firm grip is established, the rat will be gradually pulled away along a horizontal plane until it releases its grip (Takeshita et al., 2017). The maximum force exerted before grip loss will be recorded by the sensor and stored in LabChart. Each rat will undergo three independent trials with three consecutive grip strength measurements per trial. The mean force exerted across the three measurements will be averaged to determine the average grip strength per trial and the final grip strength score for each rat will be calculated as the average of the three-trial means (Takeshita et al., 2017).

#### Biomarkers

##### Irisin

Irisin, a myokine released during physical exercise, promotes neuroprotection by activating pathways such as PGC-1α/FNDC-5, which are subsequently expressed in the brain. It reduces neuroinflammation, oxidative stress and neuronal injury in models such as Alzheimer’s disease (Liu et al., 2024; Pignataro et al., 2021; Wrann et al., 2013).

##### Brain-derived neurotrophic factor (BDNF)

Exercise elevates BDNF levels through pathways mediated by irisin, enhancing synaptic plasticity and cognitive function. This is crucial in conditions like Alzheimer’s disease, where synaptic and memory impairment are prominent (Jodeiri Farshbaf and Alviña, 2021).

##### Pro brain-derived neurotrophic factor (Pro-BDNF)

Pro-brain-derived neurotrophic factor (Pro-BDNF) is the inactive precursor of mature BDNF, a critical neurotrophin in the central nervous system. Unlike mature BDNF, which enhances synaptic plasticity via TrkB receptor binding, proBDNF preferentially binds to p75∧NTR, promoting apoptosis, synaptic weakening and reduced plasticity. Elevated proBDNF levels are linked to neurodegeneration, impaired nerve regeneration and depressive-like behaviors (Barker, 2009; Wang et al., 2020; Yang et al., 2021).

##### Amyloid beta

Amyloid beta (Aβ) is a peptide produced by proteolytic cleavage of amyloid precursor protein (APP). In Alzheimer’s disease, Aβ aggregates into extracellular plaques that drive neurodegeneration through neuronal toxicity and synaptic dysfunction. These plaques further exacerbate oxidative stress by promoting reactive oxygen species (ROS) generation, leading to free radical–mediated damage and cell death (Tamagno et al., 2021; Zhang et al., 2022).

##### Myostatin

Myostatin is a well-established negative regulator of skeletal muscle growth and functional capacity. Beyond its role in muscle atrophy signaling, myostatin critically modulates muscle mass, contractile performance, and adaptive responses to exercise (Sharma and Patil, 2024). While markers like FoxO, MuRF1 and atrogin-1 offer insights into ubiquitin-proteasome pathways, they are less directly tied to measurable strength gains (Inyushkin et al., 2024; Oporto-Colicoi et al., 2025) as compared to myostatin (Muramatsu et al., 2021). Myostatin is thus prioritized as a biologically relevant, performance-correlated proxy for exercise-induced plasticity (Jang et al., 2021) complementing hypertrophy signals (IGF-1, mTOR, S6K1).

The secondary endpoint would also include changes in the structural and histological aspects of brain regions. Histological analysis is a critical component of this study as it provides direct insights into the structural and cellular changes occurring in brain tissues due to chrono-exercise interventions. Given that neurodegenerative diseases involve progressive neuronal loss, synaptic dysfunction and neuroinflammation, brain histology provides a critical means of assessing the neuroprotective effects of physical exercise.

Through histological staining techniques, we can assess neuronal survival, glial activation, and neuronal integrity, thereby elucidating the underlying mechanisms of muscle-brain communication in neurodegenerative conditions. This analysis is crucial for evaluating how structured exercise affects brain health at the cellular level and for linking neurophysiological biomarkers to functional outcomes.

The hippocampus is a critical brain structure involved in memory consolidation, spatial navigation and overall cognitive processing. It is particularly vulnerable in neurodegenerative diseases such as Alzheimer’s disease (AD) where pathological features including amyloid-beta (Aβ) accumulation, tau hyperphosphorylation, synaptic loss and neuronal degeneration are prominently observed in this region (Fischer et al., 2008; Kocahan and Doğan, 2017; Rao et al., 2022). Histological assessment of this region enables direct visualization of neuronal density, structural integrity and pathological protein accumulation, providing critical evidence of exercise-induced neuroprotection. The amygdala, a key region involved in emotional processing including anxiety, fear and affect regulation, is also strongly implicated in neurodegenerative conditions. Dysregulation of the amygdala is frequently associated with neuropsychiatric symptoms, including anxiety particularly observed in Alzheimer’s disease (Loh et al., 2023; Song, 2023). Histological analysis of the amygdala can reveal alterations in neuronal integrity, glial reactivity and protein response. This will offer a wide understanding of the neuroprotective and neuroadaptive potential of physical exercise.

## Analysis

### Enzyme-linked immunosorbent assay kit (ELISA kit)

Biomarker levels in blood plasma and brain homogenates (hippocampus and amygdala) will be measured using a commercial ELISA kit [ELK Biotechnology: cat. no. ELK9072 (irisin), cat. no. ELK5459 (BDNF), cat. no. ELK7450 (myostatin), cat. no. ELK7008, cat. no. ELK7008 (amyloid beta); FineTest Biotech: cat. no. ER1418 (Pro-BDNF)] following the manufacturer’s instructions. The assay employs a sandwich enzyme immunoassay format. ELISA was chosen for this study because it is a highly sensitive and quantitative method validated for detecting irisin, BDNF, pro-BDNF, amyloid beta and myostatin in both plasma and brain homogenates. Although Western blotting is a powerful technique for evaluating protein size and isoforms, it is semi-quantitative and more time-consuming. As the primary objective of this study is to quantify changes in protein levels rather than assess isoform-specific or post-translational modifications, ELISA represents the more appropriate choice.

For sample incubation, 100 μL of each sample will be added to the appropriate wells. Standards will be prepared by briefly centrifuging the standard vial at 7,000 rpm, then adding 1 mL of standard diluent and gently resuspending. A serial double dilution will then be prepared according to the kit protocol, and 100 μL of each dilution will be added to the designated wells. The microplate will be incubated either for 90 min at 37 °C or overnight at 4 °C to allow antigen–antibody binding.

After incubation, the sample solution will be discarded, and the wells will be washed before 100 μL of primary antibody is added to each well, followed by incubation at 37 °C for 1 h. The primary antibody solution will then be removed, and the wells will be washed three times with 250 μL of wash buffer. Subsequently, 100 μL of HRP-conjugated secondary antibody will be added to each well, and the plate will be incubated at 37 °C for 30 min. Following removal of the secondary antibody, the wells will be washed three times with 250 μL of wash buffer. Next, 90 μL of substrate solution will be added to each well and incubated for 15 min to allow color development. The reaction will be terminated by adding 50 μL of stop solution to each well, and the absorbance at 450 nm will be measured using a microplate reader (Biobase, China).

ELISA data will be analyzed using MyAssay software with a four-parameter logistic (4PL) model to determine sample concentrations. Internal quality control procedures will be implemented, including the use of positive and negative control samples to evaluate assay performance. Same-day testing will be conducted to minimize variability related to sample stability and environmental conditions. In addition, multiple replicates will be performed to enhance the reliability and statistical robustness of the comparisons (Loh et al., 2023; Thompson and Chesher, 2018).

### Fixation and sectioning

Three rats (*n* = 3) from each group will be randomly selected and their entire brains will be harvested and preserved where they will be carefully trimmed, cleaned and fixed for 24 h at 4 °C in a 4% formalin solution (Chemiz, Malaysia: cat. no.23655) (Sampedro-Carrillo, 2022). Once fixation is complete, the brain will be immersed in a 70% alcohol solution and stored at 4 °C until it sinks to the bottom of the sample vial. The tissue processing followed by dehydration through a graded ethanol series consisting of 70% ethanol (Leica Biosystems; cat. no. 3803677) (4 h), 95% ethanol (Leica Biosystems; cat. no. 3803682) (3 h) and absolute ethanol (Leica Biosystems; cat. no. 3803686) (3 h) and will subsequently be cleared in xylene (Leica Biosystem, United States: cat. no. 3803665) for 3 h. The brain tissue will be embedded in paraffin and sliced to a standardized thickness of 4.0–4.5 μm to ensure consistency across samples (Feldman and Wolfe, 2014; Sampedro-Carrillo, 2022). This will allow improved visualization of neuronal morphology and biomarker expression while minimizing variability in staining intensity.

### Staining

#### Nissl staining

Nissl staining will be performed using a commercial Cresyl Violet staining kit (Chemiz, Malaysia; cat. no. 34101) according to the manufacturer’s protocol to visualize neuronal structures, particularly Nissl bodies associated with rough endoplasmic reticulum and ribosomal RNA. Briefly, paraffin-embedded brain sections will be fixed in 4% paraformaldehyde, deparaffinized in xylene (Leica Biosystems, United States; cat. no. 3803665) and rehydrated through graded ethanol solutions (100%, 95%, 70%). After rinsing in distilled water, sections will be incubated in the Cresyl Violet staining solution for 35 min at room temperature (or 50 °C to enhance penetration), followed by differentiation in 95% ethanol under microscopic control to retain staining in nuclei and Nissl bodies. Sections will then be dehydrated in absolute ethanol, cleared in xylene (Leica Biosystem, United States: cat. no. 3803665) and mounted. Staining quality will be confirmed by the presence of blue-purple Nissl bodies and intact neuronal morphology under light microscopy. The expected results will show purple-blue neuronal cell bodies reflecting ribosomal RNA content, with nuclear staining intensity varying according to the degree of differentiation (Fedchenko and Reifenrath, 2014; Feldman and Wolfe, 2014; Hawes et al., 2009; Jacques et al., 2018; Oltmer et al., 2023). Neuronal counts will be based on clearly defined anatomical landmarks within the desired brain region and only neurons with intact cell bodies and visible nucleoli will be included.

#### Immunohistochemistry (IHC) staining

Immunohistochemistry staining will be performed according to the manufacturer’s protocols for the respective primary antibody kits (ElabScience Biotechnology Co., Ltd., United States; cat. no. E-IR-R220 and ELK Biotechnology; cat. no. ELK9072 [irisin]). Paraffin-embedded brain sections will be deparaffinized in xylene (Leica Biosystem, United States: cat. no. 3803665), rehydrated through graded ethanol (Leica Biosystem, United States) and subjected to antigen retrieval in citrate buffer (pH 6.0) at 95 °C –100 °C for 10–20 min. After cooling and washing in PBS, sections will be blocked with normal serum for 20–30 min to reduce non-specific binding. Primary antibodies will be applied at the recommended dilutions and incubated overnight at 4 °C. Following PBS washes, sections will be incubated with species-appropriate HRP-conjugated secondary antibodies for 30–60 min at room temperature. Visualization will be achieved using DAB chromogen and sections will be counterstained with hematoxylin, dehydrated, cleared in xylene (Leica Biosystem, United States: cat. no. 3803665) and mounted. Positive immunoreactivity will be identified as brown cytoplasmic or nuclear staining in the target cell populations under light microscopy. Negative controls will be included to confirm staining specificity. Two types of controls will be performed which are the No-antibody control where sections are processed through the entire IHC protocol without the addition of either primary or secondary antibodies to detect any intrinsic tissue background or endogenous pigment and the Secondary-only control where the sections will be incubated with secondary antibody alone, omitting the primary antibody, to evaluate potential non-specific binding of the secondary antibody. Cells will be considered positive when the staining intensity exceeds background levels, as determined by negative controls and displays characteristic subcellular localisation consistent with the target protein. The proportion of cells expressing the target protein will then be quantified (Fedchenko and Reifenrath, 2014; Feldman and Wolfe, 2014; Hawes et al., 2009; Jacques et al., 2018; Oltmer et al., 2023; Sampedro-Carrillo, 2022). Table 2 outlines the anticipated data collection framework and expected outcomes for both Nissl and IHC staining.

**TABLE 2:** Expected data collection framework and anticipated outcomes for Nissl and immunohistochemistry (IHC) staining.

Animal group (ID)	Nissl-neuronal density (cells/mm^2^)	IHC (marker X)–% positive cells	IHC (marker Y)–% positive cells	IHC (marker Z)–% positive cells
1	–	–	–	–
2	–	–	–	–
3	–	–	–	–

#### Hematoxylin–eosin (H&E) staining

The hematoxylin and eosin (H&E) staining process is a widely used technique in histology to highlight the structural details of tissue samples. The process will begin by preparing tissue sections fixed using alcohol or aldehyde-based fixatives (Feldman and Wolfe, 2014). The slides will then be hydrated by immersing them in water for 30 s, a critical step to prevent hematoxylin from precipitating due to interaction with salts or buffers. After hydration, the tissue will be stained with hematoxylin (Leica Biosystem, United States: cat. no. 3801576) for 30 s followed by a 1-min rinse in water and bluing agent. This step will allow for the evaluation of staining intensity and additional hematoxylin applications can be made if a deeper nuclear stain is targeted.

Following hematoxylin staining, the slides will be treated with a 1% eosin solution (Leica Biosystem, United States: cat. no. 3801619) for 10–30 s to stain the cytoplasm and other tissue structures. The tissue sections will then be dehydrated by sequential immersion in 95% and 100% alcohol each for 30 s. After dehydration, the sections will be cleared with two changes of xylene (Leica Biosystem, United States: cat. no. 3803665), which will help make the tissue transparent and prepare it for mounting. Finally, one to two drops of mounting medium, such as Dibutylphthalate Polystyrene Xylene (DPX), will be applied to each slide and a coverslip will be placed over the tissue section to preserve the stain for long-term microscopic examination. This procedure will yield blue-stained nuclei and pink-stained cytoplasmic and extracellular components allowing detailed structural visualization of the tissue (Feldman and Wolfe, 2014).

### Statistical analysis

All statistical analyses will be performed using GraphPad Prism version 8 (GraphPad Software Inc., San Diego, CA, United States). Data will be assessed for normality using the Shapiro–Wilk test and homogeneity of variance using Levene’s test. Results will be expressed as mean ± standard error of the mean (SEM), and statistical significance will be set at *p* < 0.05 (two-tailed).

For outcomes measured repeatedly over time (behavioral performance and grip strength), the primary analysis will be conducted using linear mixed-effects models (LMMs). Models will include fixed effects for exercise type, exercise timing (ZT1 vs. ZT13), treatment condition (AlCl_3_ vs control), time, and their interactions, with individual animals included as random effects to account for within-subject correlations. The group × time interaction will be used to evaluate differential treatment effects over time. This approach corresponds to the repeated-measures ANOVA framework used for G*Power sample size estimation, while providing greater flexibility in handling missing data and relaxing sphericity assumptions (Guo et al., 2013; Hilbert et al., 2019).

Model assumptions will be evaluated using residual diagnostics. When necessary, logarithmic or Box–Cox transformations will be applied. If violations persist, robust standard errors or bootstrapped confidence intervals will be used. For non-Gaussian outcomes, generalized linear mixed-effects models with appropriate link functions will be fitted and non-parametric tests will be performed only as sensitivity analyses. For endpoints measured at a single time point (plasma and brain biomarkers, neuronal counts and histological measurements), three-way ANOVA will be used to study the effects of exercise type, exercise timing, and treatment condition followed by Tukey’s HSD *post-hoc* tests when significant effects are detected.

For histological analyses, measurements obtained from multiple brain sections will be averaged to produce a single value per animal, ensuring that the individual animal remains the statistical unit of analysis. Quantitative results will be complemented by descriptive evaluation of histological features in the hippocampus and amygdala.

### Bias control and ethical consideration

Multiple methodological cautions will be implemented throughout the study to ensure the reliability and validity of the findings. Rats will be randomly assigned to the respective groups to minimize selection bias and ensure that observed outcomes are attributable to the experimental conditions. A double-blind design will be adopted to mitigate observer and measurement bias. Behavioral assessments will be conducted by a researcher blinded to group assignments and video recordings of all behavioral tests will be coded using anonymised identifiers assigned by an independent team member. These identifiers will correspond to individual rat IDs without revealing treatment details. The behavioral data will be independently scored by two trained researchers, where the first will analyze performance in the open field test and novel object recognition test while the second will verify the accuracy and consistency of scoring to enhance inter-rater reliability. Regarding the selection for the histological assessment, the individual rat will be randomly selected without considering the behavioral or biochemical outcomes. All assessments regarding the histological process will be conducted under blinded conditions, as in the behavioral assessment.

Similarly, biological samples for biomarker analysis [e.g., irisin, brain-derived neurotrophic factor (BDNF)] will be coded and processed without disclosing treatment groups to the analysts. Treatment administration and outcome assessments will be conducted by separate personnel to avoid any unintentional influence on data collection or interpretation. For histological assessment, animals will be randomly selected from each experimental group without reference to their behavioral or biochemical outcomes to avoid selection bias. All procedures related to tissue processing, imaging, and quantitative analysis will be conducted under blinded conditions. The investigators performing the histological evaluations will remain unaware of group allocation throughout the assessment process, consistent with the blinding procedures applied in the behavioral and biomarker analyses.

Environmental consistency will be maintained by housing the rats under standardized conditions, including controlled temperature, humidity and a 12-h light/dark cycle. All rats will undergo a structured acclimatization period before the intervention phase to ensure physiological adaptation and minimize stress-related variability. Throughout the study, potential confounding factors, such as signs of illness, abnormal behavior, or stress responses will be regularly monitored and documented to uphold the integrity and reproducibility of the experimental results.

## Discussion

This study is designed to generate preclinical evidence supporting chrono-combined aerobic-resistance exercise as a potential non-pharmacological strategy to attenuate cognitive decline and neurodegenerative changes in the context of AlCl_3_-induced pathology. A substantial body of literature has established the neuroprotective effects of physical exercise, demonstrating improvements in synaptic plasticity, memory retention, and anxiety-related behaviors (Guo et al., 2023; Illesca-Matus et al., 2023; Mandolesi et al., 2018). These benefits are consistently reflected in enhanced performance across behavioral paradigms such as the novel object recognition (NOR) and open-field tests.

Exercise promotes neurogenesis and synaptic plasticity through the upregulation of neurotrophic factors, particularly brain-derived neurotrophic factor (BDNF) (Lin et al., 2018; Zou and Hao, 2024). Aerobic exercise increases the myokine irisin by activating the PGC-1α/FNDC5 pathway. Irisin crosses the blood–brain barrier and stimulates BDNF expression, supporting neuronal survival, CREB phosphorylation, Nrf2-mediated antioxidative responses, and suppression of neuroinflammation and microglial activation, which are key contributors to neurodegenerative progression (Choi and Balakrishnan, 2026). In contrast, resistance exercise activates the canonical PI3K/Akt/mTOR signaling pathway in skeletal muscle, enhancing protein synthesis and IGF-1 signaling, and indirectly supporting BDNF expression through reduced muscle atrophy and systemic anti-inflammatory effects (Iglesias, 2025). Together, these mechanisms suggest that combined aerobic-resistance exercise may exert complementary neuroprotective and anti-inflammatory effects.

The present study also considers exercise timing as a potential modifying factor by aligning the intervention with defined circadian phases. Exercise was implemented at two Zeitgeber times (ZT1 and ZT13), corresponding to distinct physiological states in nocturnal rodents. This design allows examination of whether exercise performed in alignment with specific circadian phases differentially influences neurobehavioral outcomes. Emerging evidence suggests that molecular and neuroplastic responses to exercise, including irisin–BDNF signaling, may vary across the circadian cycle (Inyushkin et al., 2023). Therefore, aligning exercise sessions with circadian phase may represent an additional dimension that influences the effectiveness of physical activity under neurotoxic stress. While circadian rhythms are known to influence hormonal secretion, metabolic activity, and oxidative balance, hormonal fluctuations were not directly assessed in this study. Therefore, interpretations will focus on circadian phase–dependent physiological states rather than specific endocrine mechanisms.

Methodological validity is strengthened through the implementation of randomization procedures and blinded behavioral scoring using video-based analysis. Environmental conditions, including standardized handling and acclimatization, are carefully controlled to minimize potential confounding variables and enhance internal validity. In addition to cognitive assessments, muscular function such as grip strength is evaluated. Grip strength measurement serves as to ensure that animals retain sufficient physical capacity to perform the prescribed exercise protocols. This verification is essential to confirm that any observed differences in neurobehavioral outcomes are not confounded by inadequate engagement in the intervention.

By integrating cognitive and muscle outcomes, this study advances the concept of muscle-brain crosstalk in neurodegenerative pathology. Skeletal muscle functions not only as a contractile tissue but as an endocrine organ that converts mechanical activity into systemic molecular signals. Through exercise-induced mediators, active muscle can influence neuroplasticity, oxidative balance, and neuroinflammatory regulation, positioning muscle function as a biological driver of central adaptation. Therefore, study whether time-specific exercise optimizes this muscle-derived signaling axis to preserve both neural and neuromuscular integrity under neurotoxic stress. Framed within a circadian context, chrono-combined aerobic-resistance is conceptualized as a strategically timed systemic stimulus capable of enhancing coordinated brain–muscle resilience. This approach positions exercise as a temporally targeted, non-pharmacological strategy to mitigate neurodegenerative progression. A potential limitation of this study is the exclusive inclusion of male rats. While the use of a single-sex reduces biological variability and facilitates mechanistic interpretation, it limits the translational relevance of the findings. Sex differences have been documented in neurodegenerative susceptibility, mitochondrial function, neuroinflammatory responses, and exercise-induced neuroprotection, with estrogen and other sex hormones playing modulatory roles. Consequently, the current results should be interpreted within the context of male physiology. Future investigations should include female phenotype and directly examine sex-specific responses to concurrent exercise and neurodegenerative progression to enhance the translational validity of the model. Another important consideration is the debated translational relevance of the AlCl_3_ model. Although AlCl_3_ exposure produces several neurodegenerative features including oxidative stress, neuroinflammation, mitochondrial dysfunction, and cognitive impairment, it does not fully recapitulate the multifactorial and progressive etiology of human Alzheimer’s disease. Thus, this model should be interpreted as a paradigm for toxin-induced neurodegeneration rather than as a comprehensive representation of clinical pathology. However, emerging evidence suggests that aluminum-induced oxidative stress and chronic neuroinflammation can promote phenotypes of cellular senescence in neural and glial cells. Its effects are characterized by mitochondrial dysfunction, increased reactive oxygen species, pro-inflammatory signaling, and altered cellular homeostasis. Cellular senescence is increasingly recognized as a contributing mechanism in age-related neurodegeneration and is closely linked to circadian dysregulation and impaired stress resilience. In this context, the AlCl_3_ model provides a controlled basis for examining how timed exercise interventions may modulate stress-induced senescence pathways and neurobiological vulnerability. Therefore, while acknowledging its limitations, the model remains suitable for investigating the effects of chrono-exercise on neurotoxic and pro-senescent cellular processes.

## Conclusion

This study investigates the time-specific effects of chrono-combined aerobic-resistance exercise on neurobehavioural and molecular outcomes in an AlCl_3_-induced rat model of neurodegeneration. By combining behavioral, physical, biochemical and histological assessments, these aim to clarify how exercise timing influences muscle-brain crosstalk and cognitive resilience. The findings are expected to provide preclinical evidence supporting chrono-combined aerobic-resistance as a non-pharmacological strategy for managing neurodegenerative decline and guiding circadian-based exercise interventions in the aging population

## References

[B1] Al-Sarraf H. Mouihate A. (2022). Muscle hypertrophy in a newly developed resistance exercise model for rats. *Front. Physiol.* 13:851789. 10.3389/fphys.2022.851789 35634153 PMC9136173

[B2] Antunes M. Biala G. (2012). The novel object recognition memory: Neurobiology, test procedure, and its modifications. *Cogn. Process.* 13 93–110. 10.1007/s10339-011-0430-z 22160349 PMC3332351

[B3] Asnakew S. Nealon J. Semachew Kasa A. Ayehu G. Feleke D. Aytenew T. et al. (2025). Magnitude and predictors of mild cognitive impairment among older populations in Africa: A systematic review and meta-analysis. *Transl. Psychiatry* 15:399. 10.1038/s41398-025-03620-z 41073382 PMC12514216

[B4] AVMA. (2019). AVMA may change guidance for CO_2_ euthanasia in rodents. *J. AVMA* 254:31.

[B5] AVMA. (2020). *AVMA Guidelines for the Euthanasia of Animals*, 2020 Edn. Schaumburg, IL: AVMA.

[B6] Ayala-Méndez G. Calderón V. Zuñiga-Pimentel T. Rivera-Cerecedo C. (2023). Noninvasive monitoring of blood pressure and heart rate during estrous cycle phases in normotensive Wistar-Kyoto and spontaneously hypertensive female rats. *J. Am. Assoc. Lab. Anim. Sci.* 62 267–273. 10.30802/AALAS-JAALAS-22-000081 37130700 PMC10230531

[B7] Barker P. (2009). Whither proBDNF? *Nat. Neurosci.* 12 105–106. 10.1038/nn0209-105 19172162

[B8] Becker J. Prendergast B. Liang J. (2016). Female rats are not more variable than male rats: A meta-analysis of neuroscience studies. *Biol. Sex Differ.* 7:34. 10.1186/s13293-016-0087-5 27468347 PMC4962440

[B9] Benito B. Gay-Jordi G. Serrano-Mollar A. Guasch E. Shi Y. Tardif J. et al. (2011). Cardiac arrhythmogenic remodeling in a rat model of long-term intensive exercise training. *Circulation* 123 13–22. 10.1161/CIRCULATIONAHA.110.938282 21173356

[B10] Casas-Herrero A. Anton-Rodrigo I. Zambom-Ferraresi F. Sáez de Asteasu M. L. Martinez-Velilla N. Elexpuru-Estomba J. et al. (2019). Effect of a multicomponent exercise programme (VIVIFRAIL) on functional capacity in frail community elders with cognitive decline: Study protocol for a randomized multicentre control trial. *Trials* 20:362. 10.1186/s13063-019-3426-0 31208471 PMC6580555

[B11] Charan J. Kantharia N. (2013). How to calculate sample size in animal studies? *J. Pharmacol. Pharmacother.* 4 303–306. 10.4103/0976-500X.119726 24250214 PMC3826013

[B12] Chen Z. Shin D. Chen S. Mikhail K. Hadass O. Tomlison B. et al. (2014). Histological quantitation of brain injury using whole slide imaging: A pilot validation study in mice. *PLoS One* 9:e92133. 10.1371/journal.pone.0092133 24637518 PMC3956884

[B13] Choi J. Balakrishnan R. (2026). Aerobic exercise-induced myokine irisin release: A novel strategy to promote neuroprotection and improve cognitive function. *Neural Regen. Res.* 21 306–307. 10.4103/NRR.NRR-D-24-01034 39665814 PMC12094564

[B14] Conti F. Brito Jde O. Bernardes N. Dias Dda S. Malfitano C. Morris M. et al. (2015). Positive effect of combined exercise training in a model of metabolic syndrome and menopause: Autonomic, inflammatory, and oxidative stress evaluations. *Am. J. Physiol. Regul. Integr. Comp. Physiol.* 309 R1532–R1539. 10.1152/ajpregu.00076.2015 26423710

[B15] Dayton A. Exner E. Bukowy J. Stodola T. Kurth T. Skelton M. et al. (2016). Breaking the cycle: Estrous variation does not require increased sample size in the study of female rats. *Hypertension* 68 1139–1144. 10.1161/HYPERTENSIONAHA.116.08207 27672030 PMC5104284

[B16] Di Liegro C. Schiera G. Proia P. Di Liegro I. (2019). Physical activity and brain health. *Genes* 10:720. 10.3390/genes10090720 31533339 PMC6770965

[B17] Dial M. Malek E. Neblina G. Cooper A. Vaslieva N. Frommer R. et al. (2024). Effects of time-restricted exercise on activity rhythms and exercise-induced adaptations in the heart. *Sci. Rep.* 14:146. 10.1038/s41598-023-50113-4 38168503 PMC10761674

[B18] Ehrlich A. MacGregor K. Ashcroft S. Small L. Altıntaş A. Chibalin A. et al. (2025). HIF1α mediates circadian regulation of skeletal muscle metabolism and substrate preference in response to time-of-day exercise. *Proc. Natl. Acad. Sci. U S A.* 122:e2504080122. 10.1073/pnas.2504080122 40627397 PMC12280960

[B19] Fedchenko N. Reifenrath J. (2014). Different approaches for interpretation and reporting of immunohistochemistry analysis results in the bone tissue - A review. *Diagn. Pathol.* 9:221. 10.1186/s13000-014-0221-9 25432701 PMC4260254

[B20] Feldman A. Wolfe D. (2014). Tissue processing and hematoxylin and eosin staining. *Methods Mol. Biol.* 1180 31–43. 10.1007/978-1-4939-1050-2_3 25015141

[B21] Fischer A. Jacobson K. Rose J. Zeller R. (2008). Hematoxylin and eosin staining of tissue and cell sections. *CSH Protoc.* 2008:pdb.prot4986. 10.1101/pdb.prot4986 21356829

[B22] Grabowska K. Grabowski M. Burek M. Meybohm P. Przybyła M. Barski J. et al. (2025). Exercise training alters the hippocampal expression of blood-brain barrier components and behavior of Western diet-fed female rats. *Mol. Neurobiol.* 62 9800–9816. 10.1007/s12035-025-04873-x 40164886 PMC12289782

[B23] Guo L. Yang X. Zhang Y. Xu X. Li Y. (2023). Effect of exercise on cognitive function and synaptic plasticity in Alzheimer’s disease models: A systematic review and meta-analysis. *Front. Aging Neurosci.* 14:1077732. 10.3389/fnagi.2022.1077732 36704501 PMC9872519

[B24] Guo Y. Logan H. Glueck D. Muller K. (2013). Selecting a sample size for studies with repeated measures. *BMC Med. Res. Methodol.* 13:100. 10.1186/1471-2288-13-100 23902644 PMC3734029

[B25] Hain E. Klein C. Munder T. Braun J. Riek K. Mueller S. et al. (2016). Dopaminergic neurodegeneration in the mouse is associated with decrease of viscoelasticity of substantia Nigra tissue. *PLoS One* 11:e0161179. 10.1371/journal.pone.0161179 27526042 PMC4985068

[B26] Haripersadh R. Pillay D. Rapiti N. (2022). Impact of rapid centrifugation on routine coagulation assays in South Africa. *Afr. J. Lab. Med.* 11:1901. 10.4102/ajlm.v11i1.1901 36483324 PMC9724141

[B27] Hawes D. Shi S. Dabbs D. Taylor C. Cote R. (2009). Immunohistochemistry. *Modern Surg. Pathol.* 48–70. 10.1016/B978-1-4160-3966-2.00016-3

[B28] Hickman D. (2022). Minimal exposure times for irreversible Euthanasia with carbon dioxide in mice and rats. *J. Am. Assoc. Lab. Anim. Sci.* 61 283–286. 10.30802/AALAS-JAALAS-21-000113 35414376 PMC9137289

[B29] Hilbert S. Stadler M. Lindl A. Naumann F. Buehner M. (2019). Analyzing longitudinal intervention studies with linear mixed models. *TPM - Testing* 26 101–119. 10.4473/TPM26.1.6

[B30] Iglesias P. (2025). Muscle in endocrinology: From skeletal muscle hormone regulation to Myokine secretion and its implications in endocrine-metabolic diseases. *J. Clin. Med.* 14:4490. 10.3390/jcm14134490 40648864 PMC12249830

[B31] Illesca-Matus R. Ardiles N. Munoz F. Moya P. (2023). Implications of physical exercise on episodic memory and anxiety: The role of the serotonergic system. *Int. J. Mol. Sci.* 24:11372. 10.3390/ijms241411372 37511128 PMC10379296

[B32] Inyushkin A. Poletaev V. Inyushkina E. Kalberdin I. Inyushkin A. (2024). Irisin/BDNF signaling in the muscle-brain axis and circadian system: A review. *J. Biomed. Res.* 38 1–16. 10.7555/JBR.37.20230133 38164079 PMC10818175

[B33] Inyushkin A. Poletaev V. Inyushkina E. Kalberdin I. Inyushkin A. (2023). Irisin/BDNF signaling in the muscle-brain axis and circadian system: A review. *J. Biomed. Res.* 38 1–16. 10.7555/JBR.37.20230133 38164079 PMC10818175

[B34] Jacques A. Wright A. Chaaya N. Overell A. Bergstrom H. McDonald C. et al. (2018). Functional neuronal topography: A statistical approach to micro mapping neuronal location. *Front. Neural Circuits* 12:84. 10.3389/fncir.2018.00084 30386215 PMC6198090

[B35] Jang J. Park S. Kim Y. Jung J. Lee J. Chang Y. et al. (2021). Myostatin inhibition-induced increase in muscle mass and strength was amplified by resistance exercise training, and dietary essential amino acids improved muscle quality in mice. *Nutrients* 13:1508. 10.3390/nu13051508 33947024 PMC8146053

[B36] Jia H. Huang W. Liu C. Tang S. Zhang J. Chen C. et al. (2022). Immunosenescence is a therapeutic target for frailty in older adults: A narrative review. *Ann. Transl. Med.* 10:1142. 10.21037/atm-22-4405 36388790 PMC9652526

[B37] Jodeiri Farshbaf M. Alviña K. (2021). Multiple roles in neuroprotection for the exercise derived Myokine Irisin. *Front. Aging Neurosci.* 13:649929. 10.3389/fnagi.2021.649929 33935687 PMC8086837

[B38] Ko M. Mulia G. van Rijn R. (2019). Commonly used anesthesia/Euthanasia methods for brain collection differentially impact MAPK activity in male and female C57BL/6 mice. *Front. Cell. Neurosci.* 13:96. 10.3389/fncel.2019.00096 30983972 PMC6447702

[B39] Kocahan S. Doğan Z. (2017). Mechanisms of Alzheimer’s disease pathogenesis and prevention: The brain, neural pathology, N-methyl-D-aspartate receptors, Tau protein and other risk factors. *Clin. Psychopharmacol. Neurosci.* 15 1–8. 10.9758/cpn.2017.15.1.1 28138104 PMC5290713

[B40] Kraeuter A. Guest P. Sarnyai Z. (2019). The open field test for measuring locomotor activity and anxiety-like behavior. *Methods Mol. Biol.* 1916 99–103. 10.1007/978-1-4939-8994-2_9 30535687

[B41] Krause B. Kriegel F. Rosenkranz D. Dreiack N. Tentschert J. Jungnickel H. et al. (2020). Aluminum and aluminum oxide nanomaterials uptake after oral exposure - a comparative study. *Sci. Rep.* 10:2698. 10.1038/s41598-020-59710-z 32060369 PMC7021764

[B42] Laine S. Högel H. Ishizu T. Toivanen J. Yli-Karjanmaa M. Grönroos T. et al. (2020). Effects of different exercise training protocols on gene expression of Rac1 and PAK1 in healthy rat fast- and slow-type muscles. *Front. Physiol.* 11:584661. 10.3389/fphys.2020.584661 33329033 PMC7711069

[B43] Li F. Geng X. Huber C. Stone C. Ding Y. (2020). In search of a dose: The functional and molecular effects of exercise on post-stroke rehabilitation in rats. *Front. Cell. Neurosci.* 14:186. 10.3389/fncel.2020.00186 32670026 PMC7330054

[B44] Li J. Huang H. Fan R. Hua Y. Ma W. (2023). Lipidomic analysis of brain and hippocampus from mice fed with high-fat diet and treated with fecal microbiota transplantation. *Nutr. Metab.* 20:12. 10.1186/s12986-023-00730-7 36793054 PMC9930259

[B45] Lim J. Jung T. Lee S. Park S. Kim W. Park S. et al. (2022). Evaluation of 28-day repeated oral dose toxicity of aluminum chloride in rats. *Drug Chem. Toxicol.* 45 1088–1097. 10.1080/01480545.2020.1808670 32815395

[B46] Lin T. Tsai S. Kuo Y. (2018). Physical exercise enhances neuroplasticity and delays Alzheimer’s disease. *Brain Plast.* 4 95–110. 10.3233/BPL-180073 30564549 PMC6296269

[B47] Liu L. Fang L. Duan B. Wang Y. Cui Z. Yang L. et al. (2022). Multi-hit white matter injury-induced cerebral palsy model established by perinatal lipopolysaccharide injection. *Front. Pediatr.* 10:867410. 10.3389/fped.2022.867410 35733809 PMC9207278

[B48] Liu Y. Fu X. Zhao X. Cui R. Yang W. (2024). The role of exercise-related FNDC5/irisin in depression. *Front. Pharmacol.* 15:1461995. 10.3389/fphar.2024.1461995 39484160 PMC11524886

[B49] Loh T. Markus C. Tan C. Tran M. Sethi S. Lim C. (2023). Lot-to-lot variation and verification. *Clin. Chem. Lab. Med.* 61 769–776. 10.1515/cclm-2022-1126 36420533

[B50] Lueptow L. (2017). Novel object recognition test for the investigation of learning and memory in mice. *J. Vis. Exp.* 126:55718. 10.3791/55718 28892027 PMC5614391

[B51] Mandolesi L. Polverino A. Montuori S. Foti F. Ferraioli G. Sorrentino P. et al. (2018). Effects of physical exercise on cognitive functioning and wellbeing: Biological and psychological benefits. *Front. Psychol.* 9:509. 10.3389/fpsyg.2018.00509 29755380 PMC5934999

[B52] Mishima T. Takenaka Y. Hashimoto-Hachiya A. Tanigawa Y. Suzuki N. Oishi K. et al. (2025). Time-of-day effect of high-intensity muscle contraction on mTOR signaling and protein synthesis in mice. *Sci. Rep.* 15:23702. 10.1038/s41598-025-06709-z 40610492 PMC12229473

[B53] Muramatsu H. Kuramochi T. Katada H. Ueyama A. Ruike Y. Ohmine K. et al. (2021). Novel myostatin-specific antibody enhances muscle strength in muscle disease models. *Sci. Rep.* 11:2160. 10.1038/s41598-021-81669-8 33495503 PMC7835227

[B54] Mutalib Z. Ismail M. Miskiman N. (2020). *Spatial Analysis: Ageing Population of Multi-ethnic in Rural Area, Malaysia.* Bangkok: United Nations Economic and Social Commission for Asia and the Pacific.

[B55] Nasb M. Tao W. Chen N. (2024). Alzheimer’s disease puzzle: Delving into pathogenesis hypotheses. *Aging Dis.* 15 43–73. 10.14336/AD.2023.0608 37450931 PMC10796101

[B56] Ogunlade B. Adelakun S. Agie J. (2022). Nutritional supplementation of gallic acid ameliorates Alzheimer-type hippocampal neurodegeneration and cognitive impairment induced by aluminum chloride exposure in adult Wistar rats. *Drug Chem. Toxicol.* 45 651–662. 10.1080/01480545.2020.1754849 32329360

[B57] Oltmer J. Rosenblum E. Williams E. Roy J. Llamas-Rodriguez J. Perosa V. et al. (2023). Stereology neuron counts correlate with deep learning estimates in the human hippocampal subregions. *Sci. Rep.* 13:5884. 10.1038/s41598-023-32903-y 37041300 PMC10090178

[B58] Oporto-Colicoi V. Sepúlveda-Lara A. Marzuca-Nassr G. Sepúlveda-Figueroa P. (2025). Mild cognitive impairment and Sarcopenia: Effects of resistance exercise training on neuroinflammation, cognitive performance, and structural brain changes. *Int. J. Mol. Sci.* 26:11036. 10.3390/ijms262211036 41303517 PMC12652546

[B59] Pahlavani H. (2023). Exercise therapy to prevent and treat Alzheimer’s disease. *Front. Aging Neurosci.* 15:1243869. 10.3389/fnagi.2023.1243869 37600508 PMC10436316

[B60] Paxinos G. Watson C. (2014). *The Rat Brain in Stereotaxic Coordinates*, Seventh Edn. Amsterdam: Elsevier.

[B61] Pignataro P. Dicarlo M. Zerlotin R. Zecca C. Dell’Abate M. Buccoliero C. et al. (2021). FNDC5/Irisin system in neuroinflammation and neurodegenerative diseases: Update and novel perspective. *Int. J. Mol. Sci.* 22:1605. 10.3390/ijms22041605 33562601 PMC7915567

[B62] Rao Y. Ganaraja B. Murlimanju B. Joy T. Krishnamurthy A. Agrawal A. (2022). Hippocampus and its involvement in Alzheimer’s disease: A review. *3 Biotech.* 12:55. 10.1007/s13205-022-03123-4 35116217 PMC8807768

[B63] Reger M. Hovda D. Giza C. (2009). Ontogeny of rat recognition memory measured by the novel object recognition task. *Dev. Psychobiol.* 51 672–678. 10.1002/dev.20402 19739136 PMC2956740

[B64] Sabouri M. Kordi M. Shabkhiz F. Taghibeikzadehbadr P. Geramian Z. (2020). Moderate treadmill exercise improves spatial learning and memory deficits possibly via changing PDE-5, IL-1 β and pCREB expression. *Exp. Gerontol.* 139:111056. 10.1016/j.exger.2020.111056 32791334

[B65] Sampedro-Carrillo E. (2022). Sample preparation and fixation for histology and pathology. *Methods Mol. Biol.* 2422 33–45. 10.1007/978-1-0716-1948-3_3 34859397

[B66] Sato S. Basse A. Schönke M. Chen S. Samad M. Altıntaş A. et al. (2019). Time of exercise specifies the impact on muscle metabolic pathways and systemic energy homeostasis. *Cell. Metab.* 30 92–110.e4. 10.1016/j.cmet.2019.03.013 31006592

[B67] Scisciola L. Fontanella R. Surina N. Cataldo V. Paolisso G. Barbieri M. (2021). Sarcopenia and cognitive function: Role of myokines in muscle brain cross-talk. *Life* 11:173. 10.3390/life11020173 33672427 PMC7926334

[B68] Seibenhener M. Wooten M. (2015). Use of the open field maze to measure locomotor and anxiety-like behavior in mice. *J. Vis. Exp.* 96:e52434. 10.3791/52434 25742564 PMC4354627

[B69] Sharma S. Patil A. (2024). Myostatin’s marvels: From muscle regulator to diverse implications in health and disease. *Cell. Biochem. Funct.* 42:e4106. 10.1002/cbf.4106 39140697

[B70] Shen B. Ma C. Wu G. Liu H. Chen L. Yang G. (2023). Effects of exercise on circadian rhythms in humans. *Front. Pharmacol.* 14:1282357. 10.3389/fphar.2023.1282357 37886134 PMC10598774

[B71] Sinha S. Ellis B. (2021). *Health of Older People. Oxford Textbook of Global Public Health.* Oxford: Oxford University Press.

[B72] Song J. (2023). Amygdala activity and amygdala-hippocampus connectivity: Metabolic diseases, dementia, and neuropsychiatric issues. *Biomed. Pharmacother.* 162:114647. 10.1016/j.biopha.2023.114647 37011482

[B73] Song W. Wu W. Zhao Y. Xu H. Chen G. Jin S. et al. (2023). Evidence from a meta-analysis and systematic review reveals the global prevalence of mild cognitive impairment. *Front. Aging Neurosci.* 15:1227112. 10.3389/fnagi.2023.1227112 37965493 PMC10641463

[B74] Sooryanarayana R. Sazlina S. (2020). The Malaysian national health and morbidity survey (NHMS) 2018: Older persons’ health in Malaysia. *Geriatr. Gerontol. Int.* 20 5–6. 10.1111/ggi.14112 33370857

[B75] Stuckey J. Makhija S. Reimer D. Eswaraka J. (2023). Effects of different grades of carbon dioxide on Euthanasia of mice (Mus musculus). *J. Am. Assoc. Lab. Anim. Sci.* 62 430–437. 10.30802/AALAS-JAALAS-23-000023 37758463 PMC10597336

[B76] Sugimoto T. Kuroda Y. Matsumoto N. Uchida K. Kishino Y. Saji N. et al. (2022). Cross-sectional associations of sarcopenia and its components with neuropsychological performance among memory clinic patients with mild cognitive impairment and Alzheimer’s disease. *J. Frailty Aging* 11 182–189. 10.14283/jfa.2022.3 35441196

[B77] Takeshita H. Yamamoto K. Nozato S. Inagaki T. Tsuchimochi H. Shirai M. et al. (2017). Modified forelimb grip strength test detects aging-associated physiological decline in skeletal muscle function in male mice. *Sci. Rep.* 7:42323. 10.1038/srep42323 28176863 PMC5296723

[B78] Tamagno E. Guglielmotto M. Vasciaveo V. Tabaton M. (2021). Oxidative stress and beta amyloid in Alzheimer’s disease. Which comes first: The chicken or the egg? *Antioxidants* 10:1479. 10.3390/antiox10091479 34573112 PMC8468973

[B79] Tey N. Siraj S. Kamaruzzaman S. Chin A. Tan M. Sinnappan G. et al. (2016). Aging in multi-ethnic Malaysia. *Gerontologist* 56 603–609. 10.1093/geront/gnv153 26553738

[B80] Thapa N. Kim B. Yang J. Park H. Jang M. Son H. et al. (2020). The relationship between chronotype, physical activity and the estimated risk of dementia in community-dwelling older adults. *Int. J. Environ. Res. Public Health* 17:3701. 10.3390/ijerph17103701 32456356 PMC7277473

[B81] Thompson S. Chesher D. (2018). Lot-to-lot variation. *Clin. Biochem. Rev.* 39 51–60 . 30473592 PMC6223607

[B82] United Nation. (2019). *Ageing 2019.* Available online at: https://www.un.org/en/global-issues/ageing (accessed July 7, 2024)

[B83] van Moorsel D. Hansen J. Havekes B. Scheer F. Jörgensen J. Hoeks J. et al. (2016). Demonstration of a day-night rhythm in human skeletal muscle oxidative capacity. *Mol. Metab.* 5 635–645. 10.1016/j.molmet.2016.06.012 27656401 PMC5021670

[B84] Voigt R. Forsyth C. Keshavarzian A. (2019). Circadian rhythms: A regulator of gastrointestinal health and dysfunction. *Expert. Rev. Gastroenterol. Hepatol.* 13 411–424. 10.1080/17474124.2019.1595588 30874451 PMC6533073

[B85] Wang Q. Weng H. Xu Y. Ye H. Liang Y. Wang L. et al. (2023). Anti-osteoporosis mechanism of resistance exercise in ovariectomized rats based on transcriptome analysis: A pilot study. *Front. Endocrinol.* 14:1162415. 10.3389/fendo.2023.1162415 37664852 PMC10470051

[B86] Wang X. Ma W. Wang T. Yang J. Wu Z. Liu K. et al. (2020). BDNF-TrkB and proBDNF-p75NTR/Sortilin signaling pathways are involved in mitochondria-mediated neuronal apoptosis in dorsal root ganglia after sciatic nerve transection. *CNS Neurol. Disord. Drug Targets* 19 66–82. 10.2174/1871527319666200117110056 31957620

[B87] Wrann C. White J. Salogiannnis J. Laznik-Bogoslavski D. Wu J. Ma D. et al. (2013). Exercise induces hippocampal BDNF through a PGC-1α/FNDC5 pathway. *Cell. Metab.* 18 649–659. 10.1016/j.cmet.2013.09.008 24120943 PMC3980968

[B88] Xu L. Zhu L. Zhu L. Chen D. Cai K. Liu Z. et al. (2021). Moderate exercise combined with enriched environment enhances learning and memory through BDNF/TrkB signaling pathway in rats. *Int. J. Environ. Res. Public Health* 18:8283. 10.3390/ijerph18168283 34444034 PMC8392212

[B89] Yang B. Wang L. Nie Y. Wei W. Xiong W. (2021). proBDNF expression induces apoptosis and inhibits synaptic regeneration by regulating the RhoA-JNK pathway in an in vitro post-stroke depression model. *Transl. Psychiatry* 11:578. 10.1038/s41398-021-01667-2 34759285 PMC8580986

[B90] Zhang H. Jiang X. Ma L. Wei W. Li Z. Chang S. et al. (2022). Role of Aβ in Alzheimer’s-related synaptic dysfunction. *Front. Cell. Dev. Biol.* 10:964075. 10.3389/fcell.2022.964075 36092715 PMC9459380

[B91] Zou J. Hao S. (2024). Exercise-induced neuroplasticity: A new perspective on rehabilitation for chronic low back pain. *Front. Mol. Neurosci.* 17:1407445. 10.3389/fnmol.2024.1407445 38912176 PMC11191426

