# Correction: Chrono-combined aerobic-resistance exercises as therapeutic approach to reverse neurodegeneration in rat model: a detailed protocol

**DOI:** 10.3389/fnagi.2026.1889952

**Published:** 2026-07-13

**Authors:** Muhammad Hafiz Zuhdi Fairof, Hussin Muhammad, Amirul Hafiz Ahmad Abdullah, Farah Wahida Ibrahim, Theng Choon Ooi, Nur Liana Md Nasir, Chin Long Poo, Siti Soleha Ab Dullah, Mohd Rahimi Ashraf Abd Rahman, Elda Nurafnie Ibnu Rasid, Arimi Fitri Mat Ludin, Nor Fadilah Rajab

**Affiliations:** 1 Centre for Healthy Ageing and Wellness (H-CARE), Faculty of Health Sciences, UKM, Kuala Lumpur, Malaysia; 2 Toxicology and Pharmacology Unit, Herbal Medicine Research Centre, Institute for Medical Research, National Institutes of Health, Ministry of Health, Setia Alam, Malaysia; 3 Faculty of Health Sciences, Center for Toxicology and Health Risk Studies (CORE), UKM, Kuala Lumpur, Malaysia; 4 Premier Integrated Labs Sdn. Bhd, Kuala Lumpur, Malaysia

**Keywords:** chrono-exercise, combined aerobic-resistance exercise, muscle-brain crosstalk, neurodegenerative, neuroprotection

An incorrect **Funding** statement was provided. The correct funder is the Ministry of Higher Education, Malaysia through the Fundamental Research Grant Schemes (FRGS) with grant number FRGS/1/2024/STG03/UKM/02/2 for the whole experiment and the APC was funded by the Ministry of Health Malaysia/the correct **Funding** statement reads:

The author(s) declared that financial support was received for the research and/or publication of this article. This work was supported by the Ministry of Higher Education, Malaysia, through the Fundamental Research Grant Scheme (FRGS) [grant number FRGS/1/2024/STG03/UKM/02/2]. The article processing charge (APC) was funded by the Ministry of Health Malaysia (MOH).

[One paragraph has been mistakenly added to a wrong section, “The Open Field Test (OF-T) will be used to assess exploratory behavior in novel environments and serve as an early screening tool for anxiety-related behavior in rodents (Kraeuter et al., 2019). Since AlCl3-induced neurotoxicity is known to disrupt neurochemical pathways involved in anxiety regulation, this test will help determine whether chrono-exercise interventions can mitigate anxiety-like behaviors in AlCl3-treated rats. The experiments will be conducted under a red fluorescent light source exceeding 20 watts to ensure clear video recordings. Each rat will be placed at the edge of a black square arena (90 cm x 90 cm x 38 cm), which is divided into two zones: the peripheral zone and the central zone.

The rats' activity will be recorded for 10 minutes using a camera positioned above the arena to capture their movements. Behavioral analysis will focus on scoring the frequency of entries into and crossings through the central zone, providing insight into the rats' anxiety levels. More frequent entries into the central zone typically indicate lower anxiety, while avoidance of the central zone suggests heightened anxiety (Kraeuter et al., 2019; Seibenhener & Wooten, 2015). Each rat will be carefully handled to minimize stress before testing. The rat will be held by the base of its tail and allowed to voluntarily grasp the grip strength apparatus with its forelimbs. Once a firm grip is established, the rat will be gradually pulled away along a horizontal plane until it releases its grip (Takeshita et al., 2017). The maximum force exerted before grip loss will be recorded by the sensor and stored in LabChart. Each rat will undergo three independent trials with three consecutive grip strength measurements per trial. The mean force exerted across the three measurements will be averaged to determine the average grip strength per trial and the final grip strength score for each rat will be calculated as the average of the three-trial means (Takeshita et al., 2017).”]

A correction has been made to the section [**Outcomes measure**, *Behavioral analysis*, Open field test (OF-T) (anxiety-like behaviors), Line numbers: 374–394]:

“[The open field test (OF-T) will be used to assess exploratory behavior in novel environments and serve as an early screening tool for anxiety-related behaviors in rodents (Kraeuter et al., 2019). Since AlCl_3_-induced neurotoxicity is known to disrupt neurochemical pathways involved in anxiety regulation, this test will help determine whether chrono-exercise interventions can mitigate anxiety-like behaviors in AlCl3-treated rats. The experiments will be conducted under a red fluorescent light source exceeding 20 watts to ensure clear video recordings. Each rat will be placed at the edge of a black square arena (90 cm × 90 cm × 38 cm), which is divided into two zones: the peripheral zone and the central zone.

The rats' activity will be recorded for 10 min using a camera positioned above the arena to capture their movements. Behavioral analysis will focus on scoring the frequency of entries into and crossings through the central zone, providing insight into the rats' anxiety levels. More frequent entries into the central zone typically indicate lower anxiety, while avoidance of the central zone suggests heightened anxiety (Kraeuter et al., 2019; Seibenhener and Wooten, 2015).]”

[One paragraph has been wrongly removed to one section, “To assess muscle strength, the PowerLab system will be used comprising a grip strength meter with a force sensor, a data acquisition unit and LabChart software for real-time data collection and analysis. Before testing, the grip strength meter will be calibrated according to the manufacturer's guidelines and connected to the PowerLab system via the appropriate analogue input channel. The LabChart program will be configured to collect force data from the designated channel, ensuring precise measurement of forelimb grip strength”].

A correction has been made to the section [**Outcomes measure**, *Secondary endpoint*, Grip strength test (muscle strength), Line numbers: 408–413]:

“[To assess muscle strength, the PowerLab system will be used comprising a grip strength meter with a force sensor, a data acquisition unit and LabChart software for real-time data collection and analysis. Before testing, the grip strength meter will be calibrated according to the manufacturer's guidelines and connected to the PowerLab system via the appropriate analogue input channel. The LabChart program will be configured to collect force data from the designated channel, ensuring precise measurement of forelimb grip strength.

Each rat will be carefully handled to minimize stress before testing. The rat will be held by the base of its tail and allowed to voluntarily grasp the grip strength apparatus with its forelimbs. Once a firm grip is established, the rat will be gradually pulled away along a horizontal plane until it releases its grip (Takeshita et al., 2017). The maximum force exerted before grip loss will be recorded by the sensor and stored in LabChart. Each rat will undergo three independent trials with three consecutive grip strength measurements per trial. The mean force exerted across the three measurements will be averaged to determine the average grip strength per trial and the final grip strength score for each rat will be calculated as the average of the three-trial means (Takeshita et al., 2017).]”

The original version of this article has been updated.

